# Controversies in HIV cure research

**DOI:** 10.1186/1758-2652-15-16

**Published:** 2012-03-16

**Authors:** Rowena Johnston, Françoise Barré-Sinoussi

**Affiliations:** 1 amfAR, The Foundation for AIDS Research, 120 Wall St, 13th Floor, New York, NT 10005, USA; 2 INSERM, Institut Pasteur, 25, Rue du Docteur Roux, Paris Cedex 15 75724, France

## Abstract

**Background:**

Antiretroviral therapy significantly reduces HIV viral burden and prolongs life, but does not cure HIV infection. The major scientific barrier to a cure is thought to be the persistence of the virus in cellular and/or anatomical reservoirs.

**Discussion:**

Most efforts to date, including pharmaco, immuno or gene therapy, have failed to cure patients, with the notable exception of a stem cell transplant recipient commonly known as the Berlin patient. This case has revived interest in the potential to cure HIV infection and has highlighted the need to resolve critical questions in the basic, pre-clinical and clinical research spheres as they pertain specifically to efforts to eradicate HIV from the body of an infected person (a sterilizing cure) or at least render the need for lifelong antiretroviral therapy obsolete (functional cure). This paper describes ongoing debates in each of these research spheres as they were presented and discussed at a satellite session that took place at the 6^th ^International AIDS Society Conference on HIV Pathogenesis, Treatment and Prevention in Rome in July 2011.

**Summary:**

The resolution of these debates may have important implications for the search for a cure, the most efficient ways to identify and test promising interventions, and ultimately the availability of such a cure to diverse groups of HIV patients around the world.

## Background

Despite initial optimism concerning the curative potential of highly active antiretroviral therapy (HAART) when it became available in the mid-1990s [1], subsequent laboratory findings and clinical experience revealed that HAART does not eradicate HIV infection. In well-suppressed patients, virus can be recovered from resting CD4+ T cells [2-4], even in patients who have been successfully treated for seven years or more [5]. In addition, withdrawal of HAART almost inevitably results in viral recrudescence [6].

A cure for HIV may be envisioned in different ways. A functional cure resembles disease remission, in which virus persists, but infection does not progress after treatment interruption [7], preliminary evidence for which has been reported in the VISCONTI trial [8]. A sterilizing cure requires the eradication of all HIV and latently infected cells from the body of an infected person. The main stumbling block to a cure is the ability of the virus to persist in reservoirs that are not cleared by the host immune response or antiretroviral treatment (ART).

The nature of these reservoirs of persistent viral infection is the subject of intense debate. Most agree that some fraction of virus that remains during suppressive treatment exists in a latent, transcriptionally silent state, and that ART-resistant cellular reservoirs might persist either in long-lived cells or by proliferation of infected cells [9]. However, the degree to which reservoirs are also maintained by ongoing viral replication despite HAART remains hotly contested. The argument is not simply academic: a cure will clearly require the disruption of latent infection, but, to the extent that it occurs, will also need to address ongoing replication that is refractory to current antiretroviral therapy.

These and other basic science questions could be addressed in animal models, but the challenge here concerns the lack of an animal model that recapitulates every feature of HIV infection. Mouse models, which have the advantage of relative affordability, may have utility in characterizing persistent viral reservoirs and/or evaluating curative interventions [10], but it is possible that the extensive genetic modifications required to render them susceptible to HIV infection may compromise the generalizability of any results generated. Non-human primates have the advantage of being natural hosts of SIV infection [11], yet elements of the various simian viruses, the hosts, and the interaction between these, differ sufficiently from the human experience to raise questions concerning the applicability of results generated in these animal models too.

Several strategies have garnered sufficient basic laboratory support to warrant testing in vivo. It is unclear whether such tests should proceed directly in humans, a potentially risky undertaking, but with scientifically valid results, or in animal models in which safety and possibly efficacy might be evaluated, but with results of unknown generalizability. Safety is an important consideration in the development of new interventions for all diseases, but in the context of curing HIV infection, is additionally complicated in the patient population for whom a cure might (especially initially) be tested, namely those whose infection is already well controlled by current HAART.

Those strategies currently undergoing testing include a range of pharmacological, immunological and gene therapeutic interventions. Each rests on very different premises of how a cure might best be achieved, for example, by disrupting the chromatin environment in which latent virus resides, by disrupting the immune environment that contributes to the establishment and/or persistence of reservoirs, or by depriving the virus of target cells [12]. The only precedent for routinely curing a chronic viral infection is by pharmacotherapy in the setting of the hepatitis C virus (HCV). On the other hand, the only case of an apparent cure of HIV more closely resembles a gene therapy approach [13]. It is not clear which is most promising, although a cure for HIV might well require a combination of approaches.

The presence or absence of ongoing viral replication, the merits of testing interventions in animals first or proceeding straight to humans, and the likelihood of curing HIV using pharmacotherapy versus gene therapy were each debated in a satellite session at the 6^th ^International AIDS Society Conference on HIV Pathogenesis, Treatment and Prevention held in Rome in July 2011. Approximately 150 audience members listened to and were invited to participate in each of three debates. The arguments and discussion that took place in each of three debates at this satellite session are presented here. These and other questions in each of the research spheres (basic, pre-clinical and clinical) are important ones to solve in the search for a cure for HIV. Bringing an end to the international HIV/AIDS pandemic will require novel interventions both to prevent new infections and to cure the more than 30 million people currently estimated to be living with HIV.

## Discussion

### Is there ongoing viral replication under HAART?

Since HAART was introduced in the mid-1990s, it has increased the lifespan of patients dramatically [14]. In more recent years, as the number of available drug classes, as well as individual drug potency and tolerability, has increased, many patients can expect to suppress viral load below the limit of detection in clinical practice, currently defined as 50 copies per milliliter of blood [15]. With very few exceptions, however, patients who stop taking HAART experience rapid viral rebound to levels comparable to the viral set point at the onset of infection [6]. Clearly, although nominally undetectable (but detectable by ultrasensitive assays), the virus persists in reservoirs in a form that is impervious to current antiretroviral therapy [2-5].

There are a number of ways in which reservoirs of virus might persist: in anatomical sanctuary sites where drug penetration or potency is suboptimal; as integrated but transcriptionally silent provirus, maintained in long-lived cells or by homeostatic proliferation; or by low levels of ongoing replication that are incompletely suppressed by HAART [9]. The authors of a recent paper posit that cell-to-cell spread may contribute to viral persistence despite the presence of HAART [16]. The extent to which each of these contributes to viral recrudescence is currently unclear [9].

One test of ongoing viral replication is to intensify ART regimens to determine whether viral load can be reduced beyond what is achieved with standard suppressive regimens. Several trials have been conducted in which suppressive regimens were intensified with NNRTIs, PIs, maraviroc or raltegravir [17-23]. In each case, there was no decrease in viremia or in the size of the viral reservoirs, at least as measured in the blood. These data were interpreted to indicate a lack of ongoing viral replication, and the maintenance of reservoirs of virus in long-lived cells.

Ongoing replication might also be evidenced by genetic evolution. Because HIV is known to generate mutations with each round of replication [24], studies have compared the genomes of pre-therapy virus with those present in plasma after years of therapy [25,26]. In each case, there is little evidence of divergence during therapy.

The argument was made during the debate that if there were ongoing accumulation of genetic variation, then one might expect to see divergent virus post-therapy, with or without the development of drug resistance. In fact, in some cases there is production of predominant plasma clones that do not differ significantly from pre-therapy virus. These findings together suggest a lack of ongoing viral replication and maintenance of virus in a reservoir of homeostatically proliferating, latently infected cells.

One criticism of evidence from both kinds of studies concerns the sensitivities of the assays used to detect viremia or viral sequence evolution. The possibility was raised that ongoing replication or sequence divergence may be occurring below the limits of detection by the single copy assay (SCA) or viral sequencing assays. Although SCA values are themselves generally low (1-3 copies/ml) during suppressive therapy, this amount of virus translates into a substantial residual viral load in an infected individual. If active cycles of replication take place at extremely low levels relative to persistent viremia, it may not be detectable by even the most sensitive assays. In addition, the SCA is unable to differentiate between replication competent versus incompetent virus. It was suggested that the SCA may in fact be a measure of general cell apoptosis because as cells die and release their contents, viral RNA transcripts would be detected with this assay [27]. However, if cell death were responsible for the RNA detected, one would expect HIV DNA to be present as well, but HIV DNA is generally not present in plasma [17,18].

Anatomical sanctuary sites as a source of viral rebound were also briefly considered. Tissue levels of ART are considerably lower, especially in the brain [28] and such tissues as lymph nodes [29] than those in plasma. It is possible, then, that patients might experience ongoing viral replication in tissues even when it is not observed in blood. Because of the difficulty of sampling brain tissue, cerebrospinal fluid (CSF) is taken as a surrogate, and the extent to which the presence of virus in CSF indicates ongoing viral replication is not clear. One argument against sanctuary sites as a location of ongoing replication and source of viral rebound is the ability of regimens that do not cross the blood-brain barrier very well to nonetheless suppress the virus below the limit of detection [30].

A recent study has provided intriguing evidence that there may be ongoing viral replication in at least a substantial fraction of patients. In the largest and longest treatment intensification study to date, about one-third of 69 well-suppressed patients whose HAART regimen was intensified by raltegravir experienced a transient increase in 2-LTR circle frequency after approximately two weeks [19]. Because raltegravir blocks the integration of reverse-transcribed virus, it is difficult to imagine how the accumulation of such episomes could occur in the absence of *de novo *infection events.

Several issues were raised in response to this study: it is not clear why only about one-third of patients experienced this increase in episomes; it is not clear why the times at which their levels peaked differed between patients (ranging from 0 to 12 weeks during intensification); and it is not clear what the half-life, sequence diversity and replication competence of these 2-LTR circles is. The counter-argument was that the half-life is not relevant in this context because what was measured was an increase in 2-LTR circles over baseline and not their decay. A subsequent analysis of episomal and proviral reverse transcriptase sequences found in the peripheral blood mononuclear cells of these patients revealed statistically significant compartmentalization between these two forms of DNA and the emergence of distinct genetic populations at different time points, together suggesting that episomal and proviral DNA may originate from different anatomical compartments and that there may be stochastic release of virus from reservoirs with variable pharmacological accessibility [31].

A member of the audience introduced another line of evidence, which has since been published, suggesting ongoing replication, namely the raised levels of immune activation consistently observed even in well-suppressed patients. In the context of the large raltegravir intensification study [19], there was a decrease in the activation of CD8+ T cells during intensification that increased again when raltegravir was withdrawn [32]. This finding is consistent with the observation in one study of a trend towards a decrease in unspliced HIV RNA and increased CD8+ T cell activation in the ileum in five of seven patients undergoing maraviroc intensification [20].

Much of the research that has been conducted in this arena is in blood, largely because of the ease with which it can be sampled. There was agreement that viral replication and persistence in tissues may differ significantly from what can be observed in blood, and that one of the difficulties in looking in tissues is knowing what to divide by (as opposed to volume for blood), whether that be total cell number, CD4+ T cell number, CCR5 RNA or some other measure. A member of the audience contributed to this debate by describing ongoing unpublished work in her laboratory concerning a comparison of single proviral sequences in bone marrow, gut and peripheral blood. For patients treated either during acute or chronic infection, there is no viral sequence evolution and for each patient virus in the plasma is identical to sequences in cells both pre- and post-therapy (Sarah Palmer, personal communication).

Knowing the determinants of persistent viremia will likely inform what needs to be done to cure people. The debate concluded with a concession that it is difficult to prove a negative, the absence of ongoing viral replication, and that better assays - more sensitive and well validated - as well as more extensive studies in tissues, are needed before the question of ongoing viral replication can be resolved to universal satisfaction.

### Should candidate curative interventions be tested in animal models before human testing?

In most cases, regulatory agencies in the United States and Europe require the testing of new medical interventions (drugs, devices, biologics, vaccines, etc.) in animals before progressing to human testing [33,34]. These are intended to ensure safety and to provide some preliminary support for efficacy that justifies putting humans at potential risk. However, animal studies can provide such support only to the extent that they are generalizable to humans.

The similarities between HIV and pathogenic SIV infection in macaques are extensive. Both are characterized by chronic progressive infection associated with opportunistic infections and central nervous system disease. In both, there are also instances of relatively benign cases associated with low viremia and specific MHC-I alleles. Similar kinetics of viremia are found for both HIV and SIV, characterized by a peak during acute infection followed by a dramatic decline. Furthermore, both viral infections result in vigorous, but ultimately ineffective immune responses. Key pathogenic events, such as inflammation and chronic immune activation, mucosal immune dysfunction, microbial translocation and high levels of infection of CD4+ central memory T cells, are present in both. In addition, in both cases viral replication can be suppressed by ART [11]. On this last point, however, there is a difference that is critical in the context of cure research, namely that primates often fail to reach maximal viral suppression with the current ART optimized for primates [35,36].

Differences such as this and others that may not yet be fully characterized can have serious consequences, as raised by the debate's moderator, who cited the case of TGN1412, a monoclonal antibody intended for the treatment of leukemia and arthritis. During pre-clinical evaluation in macaques, no safety concerns were noted, but clinical testing resulted in serious adverse effects [37]. It was conceded that primate models may not always perfectly anticipate outcomes in humans, but that the answer was to find better macaque models or to design experiments that are more appropriate for existing models rather than to eliminate the non-human primate (NHP) model altogether.

Despite occasional setbacks, much has been learned about AIDS in NHP studies, including the early events of virus transmission and dissemination; basic pathogenic events in tissues; and the role of the host immune response and other elements of pathogenesis using techniques that would not be possible in humans, such as cell depletion studies, repeated tissue sampling, and elective necropsy [11]. As a counter-argument, the case was made that virological assays for evaluating HIV reservoirs before and following therapeutic intervention have been established in humans and that much has been learned from ex vivo and in vitro studies humans.

Furthermore, the only reported cure of infection has occurred in a human [13], and other studies involving gene therapy and pharmacotherapy are already ongoing in humans [12]. Agents already approved to treat other conditions, such as autoimmune disease, transplantation and cancer, could be adapted for cure studies in HIV-positive subjects in attempts to reduce immune activation and inflammation or to reverse proviral latency. However, riskier interventions, such as those involving stem cells, would benefit from pre-clinical testing in NHP. In addition, moving some of the therapies currently being employed in cancer patients with limited prognosis into HIV-positive patients who are well suppressed and generally healthy requires close consideration of the risk-benefit ratio and poses ethical challenges that are not yet resolved [38].

One of the challenges in conducting NHP studies is the lack of standardization or universally applicable model. According to the research questions being posed, researchers must use different species, virus, route and dose of inoculation, treatment regimens, assays and methods, such as sample collection. For example, studying the effects of zinc finger nuclease-mediated CCR5 knockout requires the use of a R5-tropic SHIV. The use of a virus with a mac239 envelope that is known to use two or three other co-receptors would result in an uninterpretable study. While the selection of the appropriate primate model requires careful consideration, there are several models, each with its own set of characteristics, offering the potential to find a model that is applicable to the research question of interest [11,39]. In any type of study designed to test a curative intervention, controlling such parameters as duration of infection and ART regimen is much easier in NHPs than humans.

Finally, due largely to cost, there are small numbers of animals for most NHP studies, making interpretation and reaching statistical significance difficult. A member of the panel asked for clarification of the costs of NHP studies by stating, for the sake of comparison, that even a very intensive 24-week human study, with multiple tissue biopsies, including even apheresis, would cost $15,000-$20,000 per subject, including the cost of the drug being tested (Steven Deeks, personal communication). It was conceded that NHP studies cost considerably more, at least $20,000-$30,000 per animal.

A member of the audience raised a possibly less costly alternative to humans or NHPs, namely humanized mice. He claimed that all of the interventions used in humans can be used and that because human cells are present, standard immunological measures could be used (Victor García, personal communication). It was agreed that humanized mice have some interesting features that are sufficient to warrant their use in a number of situations, but not necessarily as an alternative to NHPs.

### Is an HIV cure more likely to consist of pharmacotherapy or gene therapy?

The only precedent for routinely curing a chronic viral infection is by pharmacotherapy in the setting of HCV. On the other hand, the only case of an apparent cure of HIV more closely resembles a gene therapy approach [13]. Clinical trials are currently ongoing, testing each of these approaches, as well as immunotherapy [12].

There was disagreement about whether HIV is a genetic disease and whether gene therapy is therefore suitable as a curative strategy. Whether one of the main co-receptors for HIV that permits infection to take place, namely CCR5, or the integrated virus itself is considered, it was argued that HIV can be thought of as a genetic disease. Consistent with this philosophy, the argument was made that the Berlin patient had a genotype (heterozygous for the CCR5 dela-32 mutation) that permitted HIV infection and was cured because of a bone marrow transplant from a donor homozygous for that mutation.

Although *curing *viral infections is not commonplace, current medical treatment is dominated by pharmacotherapy and, as such, much is known about its testing and implementation. In fact, several pharmacotherapeutic interventions, including drugs that activate latent HIV (including histone deacetylase (HDAC) or methylation inhibitors, cytokines and disulfiram) or immune modulators (antibiotic, anti-rheumatologic, anti-PD-1 and protein kinase C (PKC) modulators), show promise in in vitro or animal models of latency [40], and are in clinical development for the treatment of non-HIV conditions [12]. The advantage of pursuing these pharmacotherapeutic strategies is that their safety profile is fairly well understood and they have the potential to proceed rapidly from bench to bedside.

On the other hand, trials of gene therapy are ongoing, using zinc finger nucleases (ZFNs) to excise CCR5 from CD4+ T cells ex vivo [12]. To date, no safety signals have been noted in these trials in which genetically modified autologous cells are reinfused into patients [41,42]. Safety is a key concern for gene therapy, with a history of significant toxicity and even mortality in the setting of other diseases [43]. Notably, much is already known about the apparent safety of deleting CCR5 because of the existence of a small group of people with a mutation consisting of a 32-bp deletion that leaves them without a functional CCR5 protein [44]. Still, pharmacotherapy-induced toxicities are easier to reverse than those resulting from gene therapy.

Balancing safety and potency has been difficult to date, especially with pharmacotherapeutic agents designed to reverse transcriptional latency [40]. One solution would be to combine several agents either within or between classes of mode of action. For example, multiple HDAC inhibitors, or combinations of HDAC inhibitors with immune modulation and possibly even ART intensification will quite possibly be more effective than any single agent alone. Similarly, there are several different gene therapy strategies currently under investigation that might lend themselves to combination. These include: the choice of target cells, such as T cells versus stem cells (the latter of which could then replenish all lineages of the hematopoietic system); the choice of technology, for example, targeting enzymes or siRNA; and the choice of target genetic material, such as CCR5 or the integrated virus itself [45].

Specificity is also a difficulty faced in very different ways by both pharmacotherapy and gene therapy. Without a means of identifying and therefore specifically targeting only latently infected cells, it is difficult to envision how pharmacotherapy would not affect bystander cells in potentially deleterious ways. Additionally, therapies designed to modify epigenetic processes will a priori affect cellular genes in unintended and potentially harmful ways. These side effects may be transient or reversible, but possibly at the cost of efficacy. Gene therapy is a way to target very specific processes or elements that are essential to the viral life cycle, but is only as specific as the technology in question.

There is evidence that ZFNs designed to target CCR5 may not be 100% specific to the intended DNA sequence [46], and the safety consequences of cleaving unintended targets are unknown and very possibly serious. On the other hand, because a homing endonuclease recognizes a 22 bp sequence, the likelihood of that recognition site occurring by random chance is orders of magnitude less than the number of nucleotides occurring in the human genome, suggesting that these enzymes at least have the requisite specificity. Another potential challenge for gene therapy relates to the relatively low rate of cell transformation, and for both gene therapy and some types of pharmacotherapy, such as HDAC inhibitors or PKC modulators, there is a risk of the development of cancer.

It currently seems that pharmacotherapy would be more scalable, deliverable and cost effective than gene therapy, especially because the majority of HIV-infected individuals live in regions of the world where the delivery of even currently available ART presents a daunting challenge. However, if gene therapy provided a reliable cure, then scalable delivery options may well be developed.

## Summary

Optimism regarding the potential to cure HIV infection has waxed and waned during the course of the epidemic. Several factors have contributed to the recently renewed enthusiasm regarding the possibility of a cure for HIV infection: an increasing understanding of the mechanisms of viral persistence; a growing array of therapeutic tools with the potential to cure HIV; targeted research funding directed to the search for a functional or sterilizing cure; and, not least, the first documented case of a cure of HIV infection in the Berlin patient [13], as well as preliminary evidence of a functional cure in patients who initiated ART early in infection and maintained control of infection several years after treatment discontinuation [8].

Although much has been learned concerning the mechanisms whereby virus persists in the face of HAART and a vigorous immune response, and there is general agreement about several of these mechanisms, one issue awaiting resolution is the extent to which there is ongoing viral replication [9]. This is an important issue that may shape cure strategies, especially those based on reactivating the expression of latent viral infection.

Animal models have so far been relatively under-utilized in the search for a cure, in part because of the lack of clarity regarding the applicability of the available models. Further research will likely shed light on those elements of mouse and NHP models that need optimization, although one ongoing challenge is the high cost associated with the use of NHP. As in other areas of biomedical research though, animal models provide the opportunity to address questions that would not be possible in humans.

Several concepts are already in clinical trials and are predicated on different concepts of how a cure might best be achieved. These encompass pharmacotherapy, gene therapy and immunotherapy [12]. Each has significant advantages and potential drawbacks in terms of safety, specificity and acceptability. Although pharmacotherapy is currently easier to envision as an affordable and more widely deliverable solution, advances in gene therapy are progressing rapidly and there is intense interest in simplifying its execution and delivery.

While not the explicit topic of any of the debates in this session, it is clear that several ethical issues also require resolution, such as the level of risk that is acceptable for a curative intervention in patients who are otherwise relatively healthy and well suppressed on HAART [38], as well as the deliverability of a cure to those who need it, most of whom live in resource-poor settings. However, given the enormous challenge of providing lifelong HAART to the more than 30 million people currently living with HIV [47], a cure will provide an important contribution to the goal of ending the international HIV/AIDS pandemic.

## Competing interests

The authors declare that they have no competing interests.

## Authors' contributions

RJ and FB-S designed and co-chaired the conference satellite session on which this paper is based, and co-authored this paper. Both authors have read and approved the final version of this manuscript.
